# Cytotoxicity and Apoptosis Induction of Coumarins and Carbazole Alkaloids from *Clausena Harmandiana*

**DOI:** 10.3390/molecules24183385

**Published:** 2019-09-18

**Authors:** Porntip Jantamat, Natthida Weerapreeyakul, Ploenthip Puthongking

**Affiliations:** 1 Chemistry and Natural Products Program, Graduate School, Khon Kaen University, Khon Kaen 40002, Thailand; porntipjantamat@gmail.com; 2 Division of Pharmaceutical Chemistry, Faculty of Pharmaceutical Sciences, Khon Kaen University, Khon Kaen 40002, Thailand; pploenthip@kku.ac.th; 3 Human High Performance and Health Promotion Research Institute (HHP and HP), Khon Kaen University, Khon Kaen 40002, Thailand

**Keywords:** cytotoxicity, antioxidant, apoptosis, carbzole alkaloids, coumarins, *Clausena harmandiana*

## Abstract

Seven compounds, carbazole alkaloids (heptaphylline, 7-methoxyheptaphylline, 7-methoxymukonal) and coumarins (clausarin, dentatin, nordentatin, and xanthoxyletin), were isolated from the root bark of *Clausena harmandiana*. Antioxidation, cytotoxicity and apoptosis induction were evaluated in vitro. Results showed that clausarin exerted the highest DPPH radical scavenging and 7-methoxymukonal had the highest ferric reducing antioxidant power. In contrary, dentatin was the least DPPH radical scavenger, and heptaphylline was the least reducing antioxidant power. The isolated compounds showed different cytotoxicity. The hepatocellular carcinoma (HepG2) was generally more sensitive to the isolated compounds than lung cancer (SK-LU-1), colon cancer (HCT-116), and noncancerous (Vero) cell lines, respectively. Clausarin possessed the highest cytotoxicity selectively against cancer cell lines tested. 7-Methoxymukonal and 7-methoxyheptaphylline exhibited less cytotoxicity only in HepG2 cells and were inactive in the SK-LU-1 and HCT116 cells. Despite xantoxyletin possessing low antioxidant and low cytotoxic activity, it induced the highest apoptosis percentage with the lowest necrosis percentage of HepG2 cells after 24 h. In conclusion, xantoxyletin primarily show potential anticancer activity. The root bark of *C. harmandiana* is a good source of bioactive compounds or the lead for the development of new pharmaceutical agent.

## 1. Introduction

The genus *Clausena* is in the citrus family *Rutaceae* in the major flowering plants [1,2]. Plants in *Clausena* species have been reported to majorly consist of coumarins and carbazole alkaloids [3,4,5,6,7]. The acetone extract of *C. guillauminii* roots contained carbazole alkaloids such as guillauminine A, guillauminine B, and poncitrin with reported cytotoxic, antimalarial and antimycobacterial activities [6]. The stem bark of *C. excavata* Burm. F composed of prenylated coumarins, limonoid, sterol, depside and xanthone [7]. The roots of *C. lansium* had glycozolidal [4]. Carbazole alkaloids and coumarins have been identified in the roots of *C. harmandiana* [3,8,9,10,11]. 

*C. harmandiana* (Pierre) ex Guillaumin goes under the Thai vernacular name as “Song Fa” as well [1]. It is mainly distributed in a large part of Asia including Thailand [2]. Traditionally, roots, young leaves, bark and flowers of *C. harmandiata* have been prepared as a mixture with other medicinal plants for remedy of flatulence and for food poisoning. The roots are used to relieve eye-pain, headaches and fever. The leaves are used as well for cattle feeding. According to the Thai traditional medicine, the water decoction of root has been used for antiflatulence, antipyretic, headache, tonic and stomachic. In Northeastern part of Thailand, the fresh young leaves are edible as a side dish with chili paste, while the fruit and sour young shoots are eaten with Thai food—“Laap”, and bamboo soup. According to the long history of uses, phytochemical identification of *C. harmandiana* has been conducted along with their bioactivity studies. 

Carbazoles and coumarins were major compounds found in roots, root bark, stem bark, twigs and fruit of *C. harmandiana* [5,9,10,11,12,13,14] and some identified essential oila were such as α-pinene and copaene [2]. The reported activityies of carbazoles and coumarins included antioxidant activity [15], an increasing basal glucose transport in muscle cells [12], antiplasmodial activity [9], antibacterial activity [13], antifungal activity [11], inhibition of calcium signaling [16] and an immunosuppressant [17]. Recently, *C. harmandiana* was prepared as callus to produce high production of carbazole alkaloids and probed for the antioxidant activities based on total phenolic and total flavonoid contents [18].

According to global cancer statistics in 2018, lung cancer had the highest incidence and mortality rate in both genders, followed by female breast cancer, prostate cancer, and colorectal cancer for incidence and colorectal cancer, stomach cancer, and liver cancer for mortality [19]. In Thailand, high incidences of cancer (about 60%) were from cancers of breast, cervix, colorectal, liver and lung [20] which were similar to the estimated new cancer cases and deaths of both sexes in the US [21]. Therefore, cancer is a global health concern. At the present time, there is no single drug for cancer treatment because cancer can develop therapeutic resistance and there is an occurrence of severe chemotherapy-related toxicity as well. Thus, cancer treatment requires combined therapy [22]. Hence, the researches of anticancer drug discovery and development, as well as complementary and alternative medicine, have been continuously carried out. As the geographic location of Thailand offers biodiversity advantages, leading to various bioconstituents of diverse plant species, *C. harmandiana* was investigated in the present study according to its long history of traditional uses as an herbal medicine. Our study was primarily aimed to investigate the anticancer and antioxidant activities of the isolated compounds from the roots of *C. harmandiana* and structure elucidation. Two different mechanisms of antioxidation were employed by using 2,2–diphenyl–1–picrylhydrazyl (DPPH) radical scavenging activity and ferric reducing antioxidant power. The anticancer activity was evaluated from cytotoxicity and apoptosis-inducing activity―a pharmacodynamics endpoint to confirm potential cancer therapy.

## 2. Results

### 2.1. Isolation of Carbazole Alkaloids and Coumarins

**Figure 1:**
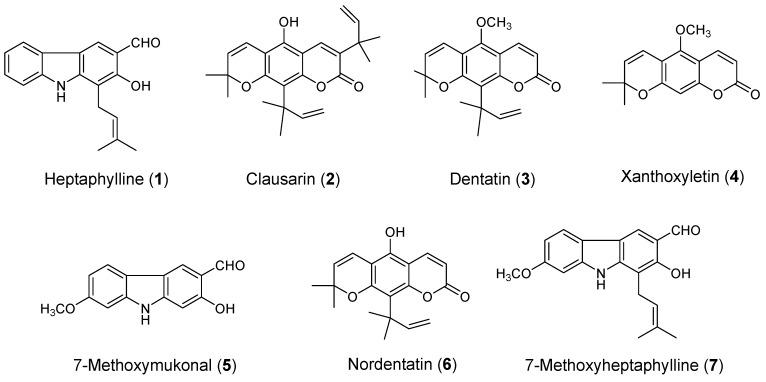
The chemical structures of carbazole alkaloids (1, 5, and 7) and coumarins (2, 3, 4, and 6).

In this study, the dichloromethane crude extract of the root bark of *C. harmandiana* (yield percentage = 6.1% dry weight) was isolated into seven knowns compounds—three carbazole alkaloids and four coumarins [23]. Carbazole alkaloids are heptaphylline **1**, 7-methoxymukonal **5**, 7-methoxyheptaphylline **7** and coumarins are clausarin **2**, dentatin **3**, xanthoxyletin **4** and nordentatin **6**. The chemical structures are shown in Figure 1. The percentage of yield of the isolated compounds (1) to (7) were 1.0%, 2.9%, 7.6%, 0.7%, 0.7%, 12.9% and 0.3% per gram of the dichloromethane extract, respectively. 

### 2.2. Antioxidant Activity

The antioxidant activity of the isolated compounds was assessed using two different mechanisms―the DPPH radical scavenging activity and the ferric reducing antioxidant power (FRAP value) (Table 1). Remarkly, clausarin exhibited the strongest antioxidant activity with the highest inhibition of DPPH radical (IC_50_ vale of 6.0 ± 0.8 µM) and high FRAP value (45.2 ± 1.0 µM FeSO_4_ equivalent). Clausarin showed higher antioxidant activity than trolox from both assays. The results of DPPH radical scavenging activity (in µg/mL) can be classified as (i) high radical scavenger: clausarin; (ii) moderate radical scavenger: trolox > 7-methoxymukonal > nordentatin; (iii) low radical scavenger: xanthoxyletin > dichloromethane crude extact > heptaphylline > 7-methoxyheptaphylline; and (iv) no activity—dentatin. 

Moreover, the reducing ability of the isolated compouds was investigated as an indicator of the potential antioxidant activity as well. The reducing power or FRAP values of the isolated compounds ranged from 1.0–47.0 μM FeSO_4_ equivalent/100 μM compound. The dichloromethane crude extract had moderate reducing ability with the FRAP value of 14.9 μM FeSO_4_/100 μg/mL extract. 7-Methoxymukonal possessed the greatest reducing ability followed by clausarin. These two compounds showed greater reducing ability than trolox. Heptaphylline showed the least reducing ability. The rank of high to low reducing ability (μM FeSO_4_ equivalent) was 7-methoxymukonal (47.0) > clausarin (45.2) > trolox (37.7) > dichloromethane crude extract (14.9) > nordentatin (9.0) > xanthoxyletin (5.2) > dentatin (4.7) > 7-methoxyheptaphylline (1.4) > heptaphylline (1.0). 

### 2.3. Cytotoxicity Activity

The cytotoxicity activity study was performed in the cancer cell lines that have never been reported―hepatocellular carcinoma (HepG2), colorectal carcinoma (HCT116) and lung adenocarcioma (SK-LU-1) cell lines in comparison to the noncancerous African green monkey kidney (Vero) cell line. The cytotoxic activity of coumarins and carbazole alkaloids were investigated comapared to cisplatin, a positive control. Results (Table 2) showed that the hepatocellular carcinoma HepG2 cell line was generally sensitive to the tested compounds based on the low IC_50_ value. The rank of high to low antiprolifertive effect in the HepG2 cell line was clausarin > cisplatin > nordentatin > heptaphylline > dentatin > 7-methoxyheptaphylline > xanthoxyletin > 7-methoxymukonal. The rank of high to low cytotoxic effect in the HCT116 cell line was clausarin > nordentatin > cisplatin > dentatin > heptaphylline > xanthoxyletin > 7-methoxyheptaphylline ≈ 7-methoxymukonal. The rank of high to low cytotoxic effect in the SK-LU-1 cell line was clausarin > cisplatin > dentatin > nordentatin > heptaphylline > xanthoxyletin > 7-methoxyheptaphylline ≈ 7-methoxymukonal. Most of the isolated compounds were inactive in noncancerous Vero cell line with the IC_50_ value higher than 100 µM—except clausarin, which showed cytotoxicity against the Vero cells with the IC_50_ value of 78.2 ± 3.0 µM. Notably, the four best cytotoxic compounds were coumarins; clausarin, nordentatin, dentatin and carbazoles; heptaphylline. Clausarin posseses the greatest cytotoxicity against all cancer cell lines—especially SK-LU-1 cell line with the lowest IC_50_ value of 6.9 ± 1.6 µM. Clausarin exerted greater cytotoxicity than cisplatin in all cancer cell lines. In contrary, 7-methoxymukonal is the least cytotoxic compound, as it was inactive in HCT116 and SK-LU-1 cell lines and had low cytotoxicity in HepG2 cell line with an IC_50_ value of 82.6 ± 2.3 µM. 

The selective index (SI) of the isolated compounds from *C. harmandiana* were determined as well, as shown in Table 2. The compound that shows the SI value higher than three, represented selective cytotoxicity to the cancer cells over the noncancerous Vero cells [24,25]. Among the isolated compounds tested, clausarin possessed the highest SI value in HepG2 cells (SI = 4.4) and in SK-LU-1 cells (SI = 11.3). Nordentatin and heptaphylline displayed the selective cytotoxicity against HepG2 cells over against Vero cells with the SI values of 3.3 and 2.6, respectively. The other isolated compounds show low selectivity (SI <3) in all cancer cell lines. Cisplatin, which is the positive control, exerted the highest cytotoxicity with high SI value in the HepG2 cells (IC_50_ = 21.8±3.0 µM and SI = 4.6). Whereas, the crude dichloromethane extract of *C. harmandiana* exhibited higher cytotoxicity and higher selectivity as well in the HepG2 cell line (IC_50_ = 25.7 ± 1.8 µg/mL and SI = 3.9) than those in the HCT116 and SK-LU-1 cell lines and was inactive in Vero cell line.

### 2.4. Determination of Apoptosis and Necrotic Cell Death

**Figure 2:**
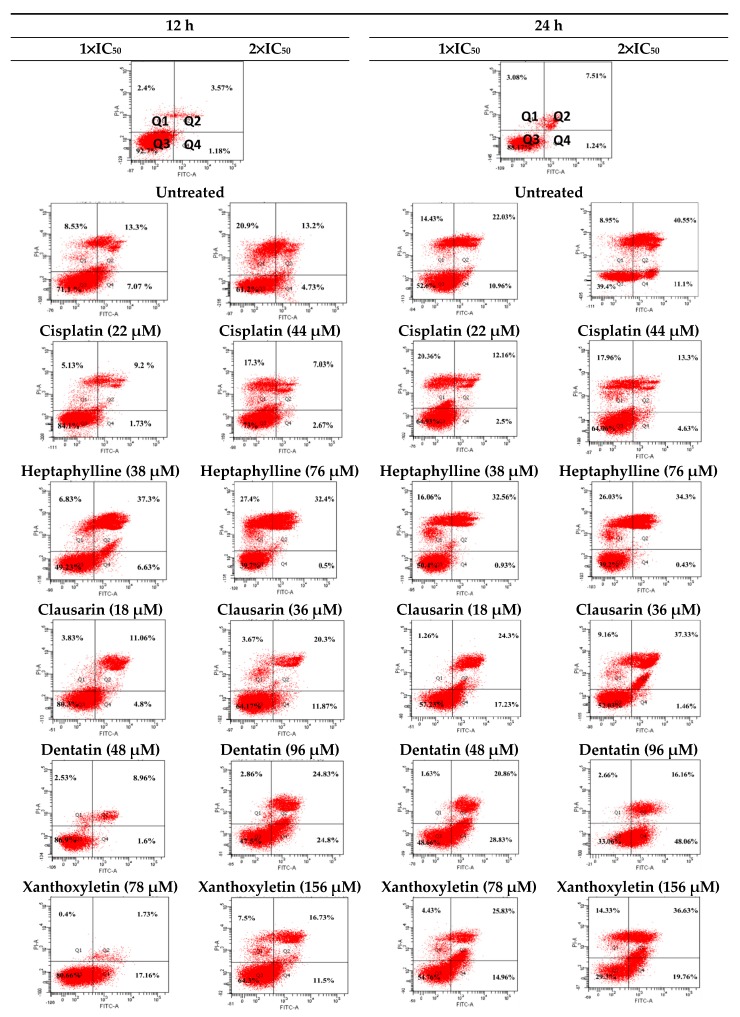

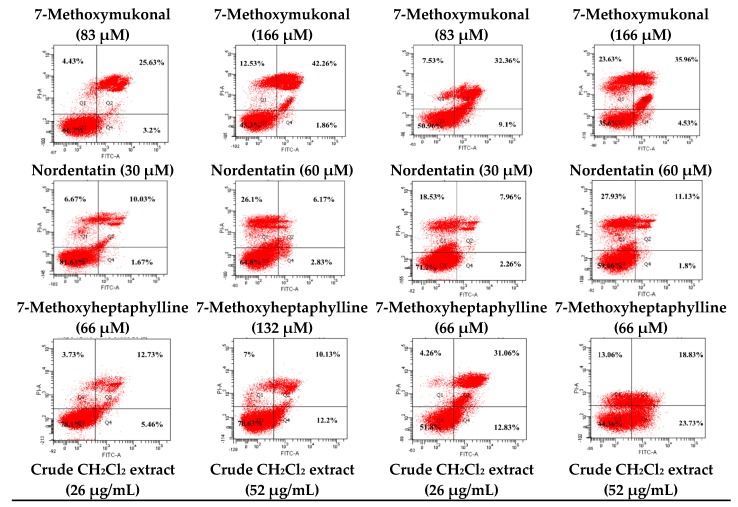
Flow cytometry histograms of the HepG2 cells after being treated with the isolated compounds and the untreated HepG 2 cells or control. Cells were treated at 1 × IC_50_ and 2 × IC_50_ concentrations for 12 and 24 h.

Due to the fact that HepG2 cell line was the most sensitive cell line in responding to the isolated compounds from *C*. *harmandiana*, it was thus selected to investigate for the induction of apoptosis and necrosis―the two major modes of cell death. The experiment was performed at two different concentrations at 1 × IC_50_ and 2 × IC_50_ for 12 and 24 h as it is important to make sure that two times the IC_50_ induce nearly 100% death of cells. The flow cytometry histograms of all treated cells and untreated cells are shown in Figure 2. 

In this study, double staining of cells by Annexin V-FITC/propidium iodide (PI) was used to differentiate apoptosis from necrosis. The stages of apoptosis can be distinguished from necrotic cell death by flow cytometry using Annexin V-FITC/PI double staining of HepG2 cells. During the early stages of apoptosis, the phosphatidylserine comprising the cell membrane will externalize to be exposed to the surface of apoptotic cells and bind with Annexin V-FITC, showing a cell dot plot in Quadrant 4. The cell dot plot is positively stained with Annexin V-FITC but negatively stained with PI (+/−). When cells are damaged or undergo a late state of apoptosis, cells will lose their membrane integrity, including nuclear membrane integrity, so that PI can pass through the nuclear membrane, showing a cell dot plot in Quadrant 2. The cell dot plot shows positively double-stained with both Annexin V-FITC and PI (+/+). During cell undergo necrotic cell death, cells are stained and appear in Quadrant 1 (−/+). Normal cells are not stained and appear in Quadrant 3 (−/−). The total apoptotic cell death percentage was counted from the percentage of cells undergoing an early state and late state of apoptosis gathering from Quadrants 4 and 2, while the necrotic cell death percentage was counted from Quadrant 1, as shown in Figure 2. 

**Figure 3:**
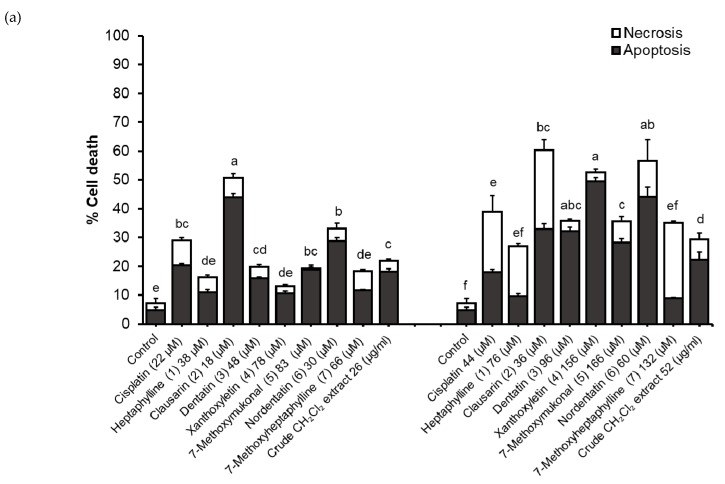

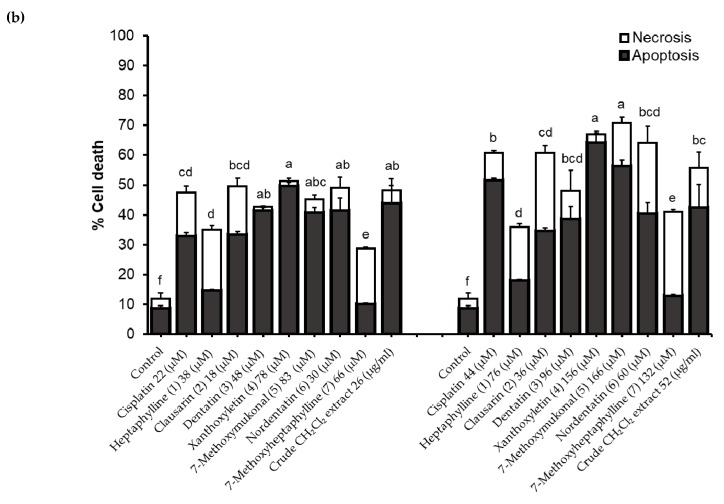
Apoptosis (■) and necrosis (□) death modes induced by the isolated compounds and crude dichloromethane extract from *C. harmandiana* at 1 × IC_50_ and 2 × IC_50_ concentrations against HepG2 cell line at (**a**) 12 h and (**b**) 24 h. Control is the untreated cells and cisplatin was used as a positive chemotherapeutic drug. The apoptotic cell percentage in each treatment groups are expressed as the mean ± SD of three experiments and were analyzed by Kruskal Wallis nonparametric statistics. Different lower-case letters indicate a significant difference of apoptosis percentage induced between compounds (*p* < 0.05).

Results showed that the HepG2 cells treated with the isolated compounds and the dichloromethane extract from roots of *C. harmandiana* underwent apoptosis and necrosis more than the untreated cells or the control (Figure 3). Generally, almost all of the isolated compounds induced apoptosis in a concentration and time dependent manners. However, apoptotic and nectrotic percentage cell deaths were varied among the isolated compounds, as shown in Figure 3.

At 12 h and 1 × IC_50_ concentration, clausarin induced the HepG2 cells to undergo the highest apoptotic cell death (44.0 ± 1.2%). However, less apoptosis was observed in cells treated with clausarin at 2 × IC_50_ concentration_._ Instead, xanthoxyletin at 2×IC_50_ concentration significantly induced the highest apoptosis percentage (49.6 ± 1.2%). Interestingly, xanthoxyletin at 1 × IC_50_ and 2 × IC_50_ concentrations caused low necrosis percentage (2.5 ± 0.7% and 2.9 ± 1.2%, respectively) than clausarin (6.8 ± 1.4% and 27.4 ± 3.8%) at 1 × IC_50_ and 2 × IC_50_ concentrations. At 24 h, xanthoxyletin induced the greatest apoptosis percentage at 1 × IC_50_ and 2 × IC_50_ concentrations as well (49.7 ± 1.1% and 64.2 ± 3.2%, respectively) with low necrotic cell death (1.6 ± 0.9% and 2.7 ± 1.1%, respectively). The apoptosis induced by xanthoxyletin was higher than cisplatin. In contrast, heptaphylline and 7-methoxyheptaphylline are the two compounds that at both concentrations induced the least apoptosis percentage at 12 and 24 h when compared to the other compounds and crude extract. 

## 3. Discussion

The presence of seven known carbazole alkaloids and coumarins in the root of *C. harmandiana* was confirmed in this study as previous reports [3,8,9,10,11]. Roots of *C. harmandiana* were previously reported to consist of (i) carbazoles, including claurailas A to D, heptaphylline, 7-methoxyheptaphylline, girinimbine, 3-formyl-1-hydroxy-7-methoxycarbazole, *o*-demethylmurrayanine, 7-hydroxyheptaphylline, murrayanine, 7-methoxymurrayanine, clausine-C, clausine E, clausine O, clausine K, clausine-V, lansine, methyl carbazole-3-carboxylate, 7-methoxymukonal, heptazoline, glycosinine, clauszoline-K and docosyl ferulate [5,9,10], clauraila E [11]; and (ii) coumarins including clausarin, xanthoxyletin, dentatin and nordentatin [5,9,10]. The root bark contained heptaphylline, clausarin, dentatin, xanthoxylectin, nordentatin, osthol [3], 2-hydroxy-3-formyl-7-methoxycarbazole and 7-methoxyheptaphylline [8].

Various bioactivities of compounds isolated from the roots of *C. harmandiana* were previously described, including antioxidant and cytotoxic activity. 7-Hydroxyheptaphylline and nordentatin were previously shown to have antioxidant activity based on DPPH assay and lipid peroxidation assay [15]. Our study showed that clausarin was the strongest antioxidant capacity based on DPPH radical scavenging activity and reducing activity, while 7-methoxyheptaphylline and nordentatin showed low and moderate antioxidant capacity, respectively. In addition, the anticancer activity was previously screened, however, in different cancer cell models from the present study. Heptaphylline and 7-methoxyheptaphylline previously exhibited strong cytotoxicity against lung cancer NCI-H187 cells, cervical cancer KB cells [5], cholangiocarcinoma KKU-OCA17 cells and KKU-214 cells [15]. Nordentatin exerted cytotoxicity against cholangiocarcinoma KKU-OCA17 cells and KKU-214 cells [15]. 7-Methoxymukonal demonstrated moderate cytotoxicity against breast cancer MCF-7 cell line [10]. Heptaphylline was the only isolated compound that was previously reported to induce apoptotic cell death in human colon adenocarcinoma cells (HT-29 cell line) [26]. It should be noted that our study compared the cytotoxicity of seven isolated compounds in the same cancer cell models, and the rank of the cytotoxicity could thus be obtained and comparatively determined. Here the cytotoxic effect was shown to be correlated with the antioxidant capacity based on DPPH and FRAP assays. Clausarin, which possessed the strongest antioxidant capacity, illustrates the greatest cytotoxicity in three cancer cell lines tested. 

Apoptosis and necrosis are among several forms of cell death mechanisms that are involved in the elimination of cancer cells, leading to successful therapy [27]. Loss of apoptosis is commonly found in most of the drug-resistant cancers [28,29]. Therefore, the induction of apoptosis in the target cancer cells is the therapeutic goal for any cancer therapy. In contrast, necrosis can trigger an inflammatory response that is not efficiently cleared by macrophages [30]. Hence the drugs that eliminate cancer cells primarily through apoptosis with no or less necrosis are good cancer drug candidates due to the lack of inflammatory responses [31]. However, whether the inflammation associated with necrosis is undesirable or not, is still controversial. Necrosis is not a physiologically programmed process and is an irreversible inflammatory form of cell death. Furthermore, the increase of immunity is not necessarily the goal of anticancer drug development from the compounds that induce necrotic cell death. Since necrosis is complicate processes, it offers multiple potential targets for drug development approaches such as for the treatment of established tumors [32]. Further studies are thus required to get a better understanding of the nature of each test compounds.

In the present study, two different exposure times (12 and 24 h) were performed to determine the mode of apoptotic cell death in treated cells by using Annexin V-FITC/PI assay. Annexin V-FITC can enter the cell and bind to phosphatidylserine either on the inner leaflet of membrane or on the outer leaflet, so it might be difficult to distinguish between the two types of cell deaths in this assay. Therefore, the Annexin V-FITC/PI assay was determined as well at an earlier endpoint (12 h) so that the early signs of apoptosis could be detected. An observation of a direct increase in Annexin V-FITC/PI (+/+) with Annexin V-FITC/PI (+/−) indicates that cells undergo a late state of apoptosis, but if they are without Annexin V-FITC/PI (+/−), it indicates that cells might undergo necrosis. These observations could be confirmed from a different time course of the study, because when cells with double positive (+/+) were observed with Annexin V-FITC/PI (+/−), it is evident that the double positively stained cells come from the Annexin V-FITC/PI (+/−) population. 

The HepG2 cell death modes induced by clausarin were via low apoptosis percentage, but high necrosis percentage, and the latter death mode was increased depending on the concentration and time. Instead, xanthoxyletin, which had relatively low cytotoxicity, low DPPH radical scavenging and reducing activity could cause the death of the HepG2 cells via the highest apoptosis percentage with less necrosis percentage. The crude extact, which was mixed of many compounds, possessed low DPPH radical scavenging and reducing activity, exhibited moderate cytotoxicity and moderate apoptosis induction with less necrosis percentage. The present results demonstrated to have different bioactivity values from the previous studies, which might be due to different methodology used for the bioactivity studies. However, it is evident that the roots of *C. harmandiana* possesses pharmaceutical potential for anticancer therapy. More mechanisms of anticancer action is worth to explore for xanthoxyletin as well. 

In conclusions, the isolated compounds from the root bark of *C. Harmandiana* exerted different degree of antioxidant, cytotoxicity, and apoposis induction activity. The antioxidant activity based on DPPH radical scavenging and reducing power were well correlated with selective cytotoxicity as observed for clausarin. Moreover, the isolated compounds induced apoptotic cell death with concentration- and time-dependent manners. Further mechanisms of anticancer action is worth to explore especially for xanthoxyletin that has the highest apoptosis inducing effect. Taken together, the root bark of *C. harmandiana* is a good source of interesting bioactive compounds and is a promising source of the lead for the development of new pharmaceutical agent.

## 4. Materials and Methods 

### 4.1. Chemicals 

All organic solvent used for the extraction were from RCI Labscan (Samut Sakhon, Thailand). Trolox (6-hydroxy-2,5,7,8-tetramethylchroman-2-carboxylic acid), 1,1-diphenyl-2-picrylhydrazyl (DPPH) and 2,4,6-tripyridyl-s-triazine (TPTZ) were purchased from Aldrich Chemistry (St. Louis, MO, USA). Cisplatin was purchased from Boryung (Ansan, Korea). DMSO was purchased from (Lab-Scan). Dulbecco’s modified Eagle’s medium (DMEM), 0.25% trypsin-EDTA (1x), fetal bovine serum (FBS), penicillin, and streptomycin were purchased from GIBCO^®^ (Invitrogen, Grand Island, NY, USA). FITC-conjugated Annexin V and propidium iodide (PI) were purchased from BioLegend (San Diego, CA, USA). Neutral red (NR) was purchased from Sigma-Aldrich (Saint Louis, MO, USA). Ferrous sulfate and iron(III) chloride hexahydrate (FeCl3•6H_2_O) were purchased from Merck (Darmstadt, Germany). TLC silica gel 60 F_254_ was purchased from Merck (Darmstadt, Germany), silica gel 60 (grain fraction 0.2–0.5 mm) was purchased from Merck (Germany). The NMR spectra of each isolated compound was recorded in CDCl_3_ on a Varian Mercury Plus spectrometer operating at 400 mHz (^1^H) and at 100 mHz (^13^C) (Varian, Inc., Palo Alto, CA, USA).

### 4.2. Plant Material

The root bark of *Clausena harmandiana* was collected in the Roi Et province, Thailand on June, 2015. The voucher specimen was deposited in Faculty of Sciences, Khon Kaen University under number KKU 21145.

### 4.3. Extraction and Isolation

The dried root bark (2.29 kg) was macerated for 48 h with dichloromethane in ratio between sample and solvent of 1:2. The organic solvent was removed after filtration and evaporated under reduced pressure followed by freeze drying to obtain the crude extract 140.23 g. The dichloromethane crude extract (20 g) was subjected into column chromatography and was eluted with the gradient of hexane and ethyl acetate to give 13 fractions that were fraction 2 as heptaphylline (1) 0.201 g, fraction 4 as clausarin (2) 0.578 g, fraction 6 as dentatin (3) 1.511 g, fraction 8 was crystallized with ethyl acetate to give xanthoxyletin (4) 0.145 g, fraction 10 as 7-methoxymukonal (5) 0.143 g and fraction 12 as nordentatin (6) 2.577 g. The crude extract (105.59 g) was isolated to give 13 fractions. Fraction 3 was crystallized with methanol to give 7-methoxyheptaphylline (7) 0.343 g. All isolated compounds were structurally elucidated using ^1^H-NMR and ^13^C-NMR. These isolated compounds were identical in all respect with the authentic samples.

Heptaphylline (1). Yield: 1.01%. ^1^H NMR (400 MHz, CDCl_3_) *δ* 11.66 (s, 1H, OH), 9.92 (s, 1H, CHO), 8.21 (brs, 1H, NH), 8.05 (s, 1H, H-4), 7.98 (d, 1H, *J* = 7.69 Hz, H-5), 7.40 (m, 2H, H-7 and H-8), 7.26 (m, 1H, H-6), 5.32 (t, *J* = 7.54 Hz, 1H, H-2′), 3.64 (d, *J* = 6.84 Hz, 2H, H-1’), 1.91 (s, 3H, H-5´/H-4´), 1.77 (s, 3H, H-4´/H-5´).^13^C NMR (CDCl_3_) *δ* 195.56, 158.00, 145.21, 140.27, 134.36, 126.02, 125.49, 123.83, 121.38, 121.02, 119.96, 117.50, 115.63, 111.01, 109.21, 25.89, 23.01, 18.29.

Clausarin (2). Yield: 2.89%. ^1^H NMR (400 MHz, CDCl_3_) *δ* 8.05 (s, 1H, H-4), 7.53 (brs, 1H, H-5), 6.71 (d, 1H, *J* = 9.92 Hz, H-6), 6.26 (dd, 1H, *J* = 17.34,10.61 Hz, H-12), 6.17 (dd, 1H, *J* = 10.62,17.44 Hz, H-2´), 5.61 (d, 1H, H-7), 5.07 (s, 1H, *J* = 16.95 H-8b), 5.03 (d, 1H, *J* = 9.97 Hz, H-8´a), 4.89 (d, 1H, *J* = 17.38 Hz, H-3´b/ H-3´a), 4.82 (d, 1H, *J* = 10.57 Hz, H-3´a H-3´b),1.60 (s, 6H, CH_3_-4´a and 4´b), 1.46 (s, 6H, CH_3_-5´a and 5´b), 1.41 (s, 6H, CH_3_-9´a and 9´b). ^13^C NMR (CDCl_3_) *δ* 161.31, 155.36, 153.24, 150.27, 147.47, 145.71, 134.87, 129.23, 128.34, 116.24, 115.11, 112.06, 108.08, 106.70, 104.71, 77.91, 40.99, 40.29, 29.64, 27.41, 26.34.

Dentatin (3). Yield: 7.76%. ^1^H NMR (400 MHz, CDCl_3_) *δ* 7.90 (d, 1H, *J* = 9.64 Hz, H-4), 6.59 (1H, d, *J* = 9.93 Hz, H-6), 6.33 (dd, 1H *J =* 17.41, 10.60 Hz, H-2´), 6.22 (d, 1H, *J* = 9.63 Hz, H-3) 5.73 (d, 1H *J* = 9.92 Hz, H-7), 4.97 (d, 1H, J = 17.48 Hz, 3´b/3´a), 4.91 (d, 1H, J = 10.61 Hz, 3´a/3´b), 3.85 (s, 3H, OCH_3_), 1.66 (s, 6H, CH_3_-4´a and 4´b), 1.48 (s, 6H, 2CH_3_-5´a and 5´b). ^13^C NMR (CDCl_3_) *δ* 160.87, 156.14, 154.07, 151.35, 149.94, 139.05, 130.50, 119.30, 116.44, 111.83, 111.75, 108.30, 107.65, 77.49, 63.54, 41.29, 29.54, 27.67.

Xanthoxyletin (4). Yield: 0.73%. ^1^H NMR (400 MHz, CDCl_3_) δ 7.83 (d, 1H, *J* = 9.64 Hz, H-4), 6.54 (d, 1H, *J* = 10.26 Hz, H-6), 6.52 (s, 1H, H-10), 6.18 (d, 1H, *J* = 9.65 Hz, H-3), 5.69 (d, 1H, *J* = 10.03 Hz, H-7), 3.84 (s, 3H, OCH_3_), 1.44 (s, 1H, 6H, CH_3_-1´a and 1´b). ^13^C NMR (CDCl_3_) *δ* 161.10, 157.62, 155.62, 152.91, 138.61, 130.66, 115.86, 112.37, 111.86, 107.43, 100.85, 77.61, 63.72, 28.19.

7-Methoxymukonal (5). Yield: 0.71%. ^1^H NMR (400 MHz, CDCl_3_) *δ* 11.42 (s, 1H, OH), 9.92 (s, 1H, CHO), 8.13 (brs, 1H, NH), 8.05 (s, 1H, H-4), 7.85 (d, 1H, *J* = 8.41 Hz, H-5), 6.89 (s, 1H, H-8), 6.87 (d, 1H, *J =* 2.13 Hz, H-6), 6.89 (s, 1H, H-1), 3.90 (s, 3H, OCH_3_). ^13^C NMR (CDCl_3_) *δ* 195.36, 160.80, 159.43, 145.89, 141.68, 126.13, 120.71, 118.02, 116.99, 115.59, 109.10, 97.06, 95.83, 55.88.

Nordentatin (6). Yield: 12.89%. ^1^H NMR (400 MHz, CDCl_3_) *δ* 8.08 (d, 1H, *J* = 9.64 Hz, H-4), 6.57 (d, 1H, *J* = 9.94 Hz, H-6), 6.25 (dd, 1H *J* = 17.16, 10.85 Hz, H-2´), 6.13 (d, 1H *J* = 9.62 Hz, H-3), 5.66 (d, 1H, *J* = 9.98 Hz, H-7), 4.89 (d,1H, *J* = 17.31 Hz, H-3´b/ H-3´a), 4.82 (d,1H, *J* = 10.53 Hz, H-3´a/H-3´b), 1.61 (s, 6H, CH_3_-4´a and 4´b), 1.42 (s, 6H, CH_3_-5´a and 5´b). ^13^C NMR (CDCl_3_) *δ* 161.69, 156.10, 154.30, 150.21, 146.76, 139.43, 130.13, 116.31, 115.12, 110.39, 108.22, 106.24, 104.05, 77.25, 41.18, 29.72, 27.47.

7-Methoxyheptaphylline (7). Yield: 0.32%. ^1^H NMR (400 MHz, CDCl_3_) *δ* 11.64 (s, 1H, OH), 9.89 (s, 1H, CHO), 8.12 (brs, 1H, NH), 7.92 (s, 1H, H-4), 7.83 (d, 1H, *J* = 8.49 Hz, H-5), 6.91 (d, 1H, *J* = 1.6 Hz, H-8), 6.86 (dd, 1H, *J* = 8.5, 2.09 Hz, H-6), 5.31 (m, 1H, H-2´), 3.90 (s, 3H, OCH_3_), 3.62 (d, 2H, *J* = 6.83 Hz, H-1´), 1.90 (s, 3H, H-5´/ H-4´), 1.77 (s, 3H, H-4´/H-5´), ^13^C NMR (CDCl_3_) *δ* 195.54, 159.18, 157.45, 145.34, 141.61, 134.29, 124.20, 121.45, 120.67, 117.65, 117.39, 115.52, 109.20, 109.09, 95.78, 55.68, 25.89, 23.00, 18.29.

### 4.4. Antioxidant Activity

#### 4.4.1. DPPH Radical Scavenging Assay 

The 2,2–diphenyl–1–picrylhydrazyl (DPPH) radical scavenging assay was conducted as per the previous method [33]. Briefly, the stock solutions of 10 mM DPPH was prepared in methanol. The DPPH was mixed with samples (100 µL each) at various concentrations (1−500 µM) in 96 wells plate and incubated for 30 min in a dark condition. The absorbance of DPPH radical was measured at 517 nm using a microplate reader (Tecan, Lyon, France). Trolox was used as a positive control. The half maximum inhibitory concentration (IC_50_ value) was calculated from the plot of percentage of DDPH radical inhibition versus concentration of samples using the following formula:

% DPPH radical inhibition = [(Ab − As)/Ab] × 100,(1)

where Ab = absorbance of the reagent blank, and As = absorbance of the sample. Each experiment was done in five replications and results are expressed as average values.

#### 4.4.2. Ferric Reducing Antioxidant Power (FRAP) Assay

FRAP assay was performed following the pervious study [34] with slight modification. FRAP reagent was prepared by mixing 300 mM acetate buffer (pH 3.6), 20 mM ferric chloride solution and 10 mM TPTZ solution in the ratio of 10:1:1 and incubated at 37 °C for 30 min. Then, 150 µL of FRAP reagent was added with 50 µL of the sample (100 µM of isolated compounds or 100 µg/mL of the CH_2_Cl_2_ crude extract) in each well of the 96-wells plate. The absorbance was measured at 595 nm using microplate reader (Tecan, Lyon, France). Trolox was used as a positive control. The FRAP value was calculated from the linear equation between the absorbance and ferrous sulfate standard curves (y = 0.0548x + 0.0213, R^2^ = 0.9989) and expressed as µM of ferrous sulfate equivalents.

### 4.5. Cell Lines and Cell Culture 

The human lung adenocarcinoma cell line (SK-LU-1), human colon carcinoma cell line (HCT116), human hepatocellular carcinoma cell line (HepG2) and noncancerous African green monkey kidney epithelial cell line (Vero) were cultured in Dulbecco’s modified Eagle’s medium (DMEM) supplemented with 10% fetal bovine serum, 100 IU/mL penicillin and 100 mg/mL streptomycin. Cells were incubated at 37 °C with 95% air and 5% CO_2_ until they reached approximately 80% confluence before use. 

#### 4.5.1. Cell Viability Assay

Cell viability assay was assessed by neutral red (NR) [35]. Briefly, cells at a density of 3 × 10^4^ cells/well were seeded in 96-wells plate and incubated for 24 h. The sample at concentration, ranging from 10 to 500 µM, were added and incubated for 24 h. After incubation time, cells were washed with PBS. NR reagent (50 µg/mL) was added to each well and incubated for another 2 h. After that, cells were washed with PBS and lyzed with 0.33% HCl in isopropanol. Cisplatin was used as a positive control. The absorbance of NR was detected using microplate reader (Tecan, Lyon, France) at 520 nm. The percentage of cell viability was calculated vis-à-vis the untreated cells and cytotoxicity was expressed as the inhibition concentration at 50% (IC_50_). The final DMSO concentration was not exceed 0.1%v/v to lessen the cytotoxicity (<10%).

#### 4.5.2. Mode of Cell Death by Annexin V-FITC/PI Staining Analyzed by Flow Cytometry

The Annexin V-FITC and PI apoptosis assay kit (BioLegend, San Diego, CA, USA) was used to determine mode of cell death as per previous report [36]. The HepG2 cells (8 × 10^5^ cell/mL) were seeded in a 24-well plate and incubated for 24 h. The compounds at a concentration of 1 x IC_50_ and 2 x IC_50_ were added and incubated for 12 and 24 h. After that, cells were washed with PBS and cold BioLegend’s cell staining buffer followed by staining with Annexin V-FITC and PI and cell were incubated in the dark for 15 min. The cells were analyzed by flow cytometry (BD FACSCanto II, BD Biosciences, San Jose, CA, USA) and the percentage of cells in different populations were calculated with FACSDiva software.

### 4.6. Statistical Analysis

The data were presented as the means ± SD. Differences among samples were defined using one-way ANOVA followed by a Tukey’s multiple comparison post hoc test using SPSS 19.0 for Windows^®^ (SPSS Inc., IL, USA). The %apoptotic cells in each treatment groups are expressed as the mean ± SD of three experiments and were analyzed by Kruskal Wallis nonparametric statistics. Any differences with a *p* value of <0.05 were considered statistically significant.

## Figures and Tables

**Table 1:** Antioxidant capacities of isolated compounds and crude extract from *C. harmandiana*.

Compounds	DPPH Assay		FRAP Assay
	IC_50_ (µM)	IC_50_ (µg/mL)	FRAP Value (µM FeSO_4_ Equivalents)
Trolox	16.8 ± 0.6^b^	4.2 ± 0.1^a^	37.7 ± 0.8^c^
Heptaphylline (1)	335.1 ± 7.8^g^	93.5 ± 2.2^f^	1.0 ± 0.0^g^
Clausarin (2)	6.0 ± 0.8^a^	2.3 ± 0.3^a^	45.2 ± 1.0^b^
Dentatin (3)	>500^h^	>500^h^	4.7 ± 0.1^f^
Xanthoxyletin (4)	247.1 ± 3.0^e^	63.8 ± 0.8^d^	5.2 ± 0.3^f^
7-Methoxymukonal (5)	26.2 ± 2.0^c^	6.8 ± 0.5^b^	47.0 ± 0.6^a^
Nordentatin (6)	38.3 ± 2.5^d^	12.0 ± 0.8^c^	9.0 ± 1.1^e^
7-Methoxyheptaphylline (7)	313.4 ± 3.4^f^	96.8 ± 1.1^g^	1.4 ± 0.1^g^
Crude CH_2_Cl_2_ extract		80.5 ± 0.7^e^	14.9 ± 0.1^d^

IC_50_ >500 µM, as at the maximum concentration (500 µM), the inhibition of DPPH radical was less than 43%. Different lower-case letters in the same column indicate a significant difference between compounds (*p* < 0.05).

**Table 2:** Cytotoxicity and selectivity index of isolated compounds and CH_2_Cl_2_ crude extract from *C. harmandiana*.

Compounds	IC_50_ (µM) and (Selectivity Index)			
	HepG2	HCT116	SK-LU-1	Vero
Cisplatin	21.8 ± 3.0^aB^	71.9 ± 2.7^cC^	42.7 ± 1.9^bB^	inactive^dB^
	(4.6)	(1.4)	(2.3)	
Heptaphylline (1)	37.8 ± 2.6^aD^	74.7 ± 2.1^bC^	83.1 ± 2.6^cD^	inactive^dB^
	(2.6)	(1.3)	(1.2)	
Clausarin (2)	17.6 ± 2.1^bA^	44.9 ± 1.4^cA^	6.9 ± 1.6^aA^	78.2±3.0^dA^
	(4.4)	(1.7)	(11.3)	
Dentatin (3)	47.6 ± 2.8^aE^	73.9 ± 2.5^bC^	45.4 ± 2.5^aB^	inactive^cB^
	(2.1)	(1.4)	(2.2)	
Xanthoxyletin (4)	78.2 ± 2.2^aG^	79.8 ± 2.8^aD^	94.4 ± 2.2^bE^	inactive^cB^
	(1.3)	(1.3)	(1.1)	
7-Methoxymukonal (5)	82.6 ± 2.8^aH^	inactive^bE^	inactive^bF^	inactive^bB^
	(1.2)	(1.0)	(1.0)	
Nordentatin (6)	29.9 ± 3.2^aC^	63.6 ± 2.3^bB^	67.3 ± 1.2^bC^	inactive^cB^
	(3.3)	(1.6)	(1.5)	
7-Methoxyheptaphylline (7)	65.5 ± 2.7^aF^	inactive^bE^	inactive^bF^	inactive^bB^
	(1.5)	(1.0)	1.0)	
Crude CH_2_Cl_2_ extract (µg/mL)	25.7 ± 1.8^a^	67.9 ± 2.6^b^	67.4 ± 2.7^b^	inactive^c^
	(3.9)	(1.5)	(1.5)	

Inactive means cell viability percentage was >50% at the maximum concentration used (>100 µM for the compound or >100 µg/mL for the extract). Different lower-case letters indicate a significant difference of the compounds between cell lines and different capital letters indicate a significant difference between compounds in the same cell line (*p* < 0.05).
